# Genome-wide association mapping for seedling and adult resistance to powdery mildew in barley

**DOI:** 10.1007/s00122-024-04550-y

**Published:** 2024-02-16

**Authors:** Jie Guo, Chenchen Zhao, Sanjiv Gupta, Greg Platz, Lisle Snyman, Meixue Zhou

**Affiliations:** 1 College of Agronomy, Shanxi Agricultural University, Jinzhong, 030801 China; 2 Tasmanian Institute of Agriculture, University of Tasmania, Launceston, TAS 7250 Australia; 3 Western Crop Genetics Alliance, College of Science, Health, Engineering and Education, Murdoch University, Murdoch, 6150 Australia; 4 Department of Primary Industries and Regional Development, South Perth, WA Australia; 5 Department of Agriculture and Fisheries, Hermitage Research Facility, Warwick, QLD 4370 Australia

## Abstract

**Key message:**

Two new major QTL were identified for powdery mildew resistance. We confirmed that the QTL on 7HS contributed mainly to the adult-plant resistance, while another one on chromosome arm 1HS made a significant contribution to the seedling resistance.

**Abstract:**

Powdery mildew (PM), caused by *Blumeria hordei*, can occur at all post emergent stages of barley and constantly threatens crop production. To identify more genes for effective resistance to powdery mildew for use in breeding programs, 696 barley accessions collected from different regions of the world were evaluated for PM resistance at seedling and adult growth stages in three different states of Australia. These barley accessions were genotyped using DArTSeq with over 18,000 markers for a genome-wide association study (GWAS). Using the FarmCPU model, 54 markers showed significant associations with PM resistance scored at the seedling and adult-plant stages in different states of Australia. Another 40 markers showed tentative associations (LOD > 4.0) with resistance. These markers are distributed across all seven barley chromosomes. Most of them were grouped into eleven QTL regions, coinciding with the locations of most of the reported resistance genes. Two major MTAs were identified on chromosome arms 3HS and 5HL, with one on 3HS contributing to adult plant resistance and the one on 5HL to both seedling and adult plant resistance. An MTA on 7HS contributed mainly to the adult-plant resistance, while another one on chromosome arm 1HS made a significant contribution to the seedling resistance.

**Supplementary Information:**

The online version contains supplementary material available at 10.1007/s00122-024-04550-y.

## Introduction

Powdery mildew caused by *Blumeria hordei* is an important foliar disease of barley (*Hordeum vulgare* L.). Early powdery mildew infection in barley can cause yield loss in susceptible barley varieties of up to 25% and late infection of up to 10% (https://www.agric.wa.gov.au/barley/management-barley-powdery-mildew-face-fungicide-resistance). The disease occurs worldwide in most places where barley is grown, particularly in areas with cooler, moist climates. The use of resistant varieties is the most effective method of disease control in sustainable cropping systems minimizing both financial and labor inputs for growers.

Many powdery mildew resistance genes have been reported in barley (Ames et al. 2015; Jørgensen 1994). These genes confer seedling resistance and/or adult-plant resistance (APR). Those that function at both seedling and adult-plant stages demonstrate all stage resistance (ASR). Resistance may be complete (immunity) or incomplete (partial resistance), race-specific or durable (like *mlo* resistance). Qualitative genes condition the host resistance to avirulent pathotypes or susceptibility to virulent pathotypes of the pathogen. Nearly 100 qualitative genes have been reported and most of them are located on chromosome 1H, i.e., *Mla* alleles (Dreiseitl 2020). However, gene-for-gene resistance is frequently found in interactions between plants and host-adapted pathogens. Based on the functional mode of R genes in a gene-for-gene system (Flor 1971), in which specificity of the R gene to virulence genes from a complementary pathotype, evolving pathogens due to the use of specific R genes will eventually adapt to new varieties and overcome the resistance functions (Cowger et al. 2018; Golzar et al. 2016). Non-functional *mlo*, a nonspecific recessive gene, confers broad-spectrum resistance against an entire PM species (Dreiseitl 2020). *mlo* is widely used in European spring barley breeding programs (Dreiseitl 2017). Even though breeders have overcome the pleiotropic effects of the *mlo* genes, viz. necrotic leaf spotting and reduced grain yield, it is still recommended that *mlo* should not be used for breeding winter barley as the presence of *mlo* in both spring and winter barley could support year-round adaptation of the pathogen and result in the subsequent development of partial virulence and gradual erosion of the effectiveness of this unique resistance gene (Jørgensen 1992). Apart from the above race-specific *Mla* and non-race-specific *mlo* genes, many quantitative trait loci (QTL) spanning a genomic region, containing powdery mildew resistance genes in barley have also been reported (Ames et al. 2015; Ge et al. 2021; Gupta et al. 2018; Li and Zhou 2011; Novakazi et al. 2020; Piechota et al. 2019, 2020; Shtaya et al. 2006; Silvar et al. 2010). These QTL are distributed to all 7 chromosomes with some containing known seedling/adult-plant resistance genes. Most of these reported QTL contribute to partial resistance yet provide viable options for breeding barley resistant to PM by pyramiding quantitatively inherited resistance genes or by combining these QTL containing major effect or race-specific resistance genes.

Pyramiding several genes effective against the whole spectrum of pathotypes into one genotype can greatly increase the duration of the resistance. This strategy depends on the continuous supply of new sources of resistance including independently inherited genes to deal with evolving pathogen populations. Due to the limited number of resistance genes in crop cultivars, researchers have started “rewilding” crops (Razzaq et al. 2021) for the lost resistance or searching for new resistance genes from landrace collections. This study aims to identify potentially novel MTAs that are effective against barley powdery mildew at both seedling and adult-plant stages, using a set of 696 barley accessions collected worldwide. These barley accessions include a large proportion of landraces and wild barley collections. As the detection of MTAs, especially with minor effects, varies between environments and populations, screening of the accessions was conducted in three locations at two stages, i.e., seedling and adult-plant stages.

## Materials and methods

### Plant materials and marker genotyping

A set of 696 barley genotypes collected from different countries were used in this study. More than half of them are landraces or wild barleys. Briefly, these genotypes are composed of Chinese landraces and cultivated varieties (approximately 30%), Australian commercial varieties (5%), wild barley genotypes of different global origins (7%), and European collections (approximately 58%). The split of genotypes between two-row and six-row barley is approximately 70% and 30%, respectively. DNA was extracted from leaf tissue collected at the 2-leaf-stage seedlings from a single plant per accession and genotyped with DArTSeq (http://www.diversityarrays.com/dart-application-dartseq). Over 33,000 DArT and 31,000 SNP markers were scored. A total of 18,000 markers were used for marker-trait association study, after removing those with the same scores or with greater distortion (minor allele frequency of < 0.05) and/or a greater proportion of missing data (> 10%). The marker positions are based on Barley cv. Morex, version MorexV2 assembled by TRITEX (Monat et al 2019).

### Assessments of powdery mildew resistance

*Glasshouse test:* The evaluation of seedling resistance was conducted in pot experiments at Perth, Western Australia (WA) and Launceston, Tasmania (TAS). Barley lines were sown in 10-cm-diameter plastic pots in clumps of 10 seeds per line and two lines per pot using a pasteuriszed potting mix (two parts river sand and one part peat moss with nutrients and trace elements). Plants were grown in the glasshouse set at 18–22 °C with 16 h day/8 h night for 2 weeks when the second seedling leaf was fully unfolded. Leaves were inoculated by dusting *Bgh* conidiospores multiplied on the susceptible cultivar *Baudin*. Plants were then returned to the glasshouse for symptom development. There were two replications of each entry at both sites. At Warwick, Queensland (QLD), a detached leaf assay method was used to assess seedling resistance to *Bgh* (Dreiseitl 2022). Three primary leaf segments of each genotype were arranged across 150 mm petri dishes containing 0.8% water agar modified with benzimidazole at 40 mg/L. All petri dishes were then inoculated with a mixture of *Bgh* isolates that were collectively virulent for *Mla3*, *Mla8*, *Mla9*, *Mla12*, *Mla22*, *Mlat*, *Ml*(*Ch*), *Mlg*, *MlGa*, *Ml*(*He2*), *Mlk1*, *MlLa*, *Mlnn*, *Mlra*, and *MlRu2*. Colonies of powdery mildew were fully developed 7 d post-inoculation and were assessed on the eighth day using a modified scale of Torp et al. (1978) where 0 (no signs of disease) to 5 (very severe), i.e., 0 = immune (HR); 1 = resistant (R); 2 = moderately resistant (MR); 3 = moderately susceptible (MS); 4 = susceptible (S) and 5 = highly susceptible (HS).

*Field screening:* Field experiments were conducted in the respective states to assess powdery mildew resistance at the adult-plant stages. A plot comprised single 1-m rows (Perth and Launceston) or hill plots (Warwick). A randomized complete block design was used in Perth and Launceston experiments with two replicates of each barley line. These nurseries were surrounded by spreader rows of the powdery mildew-susceptible variety Baudin, which was infected by natural inoculum of *Bgh* before plots were sown. An additional spreader row was located approximately in the middle of each experiment. In Queensland, the disease nursery was conducted in Toowoomba. Paired rows of datum plots were sown parallel and 75 cm distant from susceptible spreader rows. Test lines were sown in entry order and not replicated. The nursery was inoculated with the same mixture of pathotypes that were used for the seedling test but did not include an *MlLa* virulent isolate; however, virulence for this gene was confirmed to be present post assessment and must have arrived through natural infection. Response to PM infection was independently assessed by two operators when most lines were at anthesis (Zadoks growth stage 60–69; Zadoks et al. 1974) using a 0–9 scale, where 0 = no symptoms of disease and 9 = highly susceptible. Plants with 0–3 ratings were classified as resistant, 4–6 as intermediate and 7 and beyond as susceptible.

### Genome-wide association analysis

Marker-trait associations (MTAs) were identified by GWAS using the rMVP package of R software (Yin et al. 2021) A general linear model (GLM), mixed linear model (MLM), and FarmCPU were used for the association tests. The FarmCPU has recently been proposed to be a superior model for GWAS (Kaler et al. 2020). In the association study, the threshold of *P* was set at < 0.0001 (–log_10_ (*P*) > 4) to indicate the significant MTAs. The Manhattan and Quantile–quantile (Q-Q) plots were drawn using the R software. The Q-Q plot was selected to evaluate the fitness and efficiency of these models. The MapQTL 6.0 software package (Van Ooijen 2009) was used to determine the locations of significant QTL based on phenotypic variations.

### Statistical analysis

ANOVA and correlation analysis were performed using GraphPad Prism version 10.0.0 for Windows, GraphPad Software, Boston, Massachusetts USA, www.graphpad.com.

### Search for putative candidate genes

Identification of candidate genes followed a simple theory targeted at discovering homologs of cloned barley PM genes that overlapped with MTAs identified in this research. To be specific, nine representative cloned genes responsible for resistance to PM in barley were selected based on an extensive literature review. Protein sequences of these genes were downloaded from the barley genome database *Gramene* (https://ensembl.gramene.org/Hordeum_vulgare) before blasting them using the tools of BLASTP (protein sequence blasting, https://ensembl.gramene.org/Tools/Blast). Blast results were then compared with the physical positions of MTAs identified. Where the positions of previously identified PM resistance genes overlapped with the QTL regions identified in this GWAS those resistance genes were deemed to be present in the germplasm screened.

## Results

### Barley seedling/adult-plant resistance to powdery mildew

**Fig. 1:**
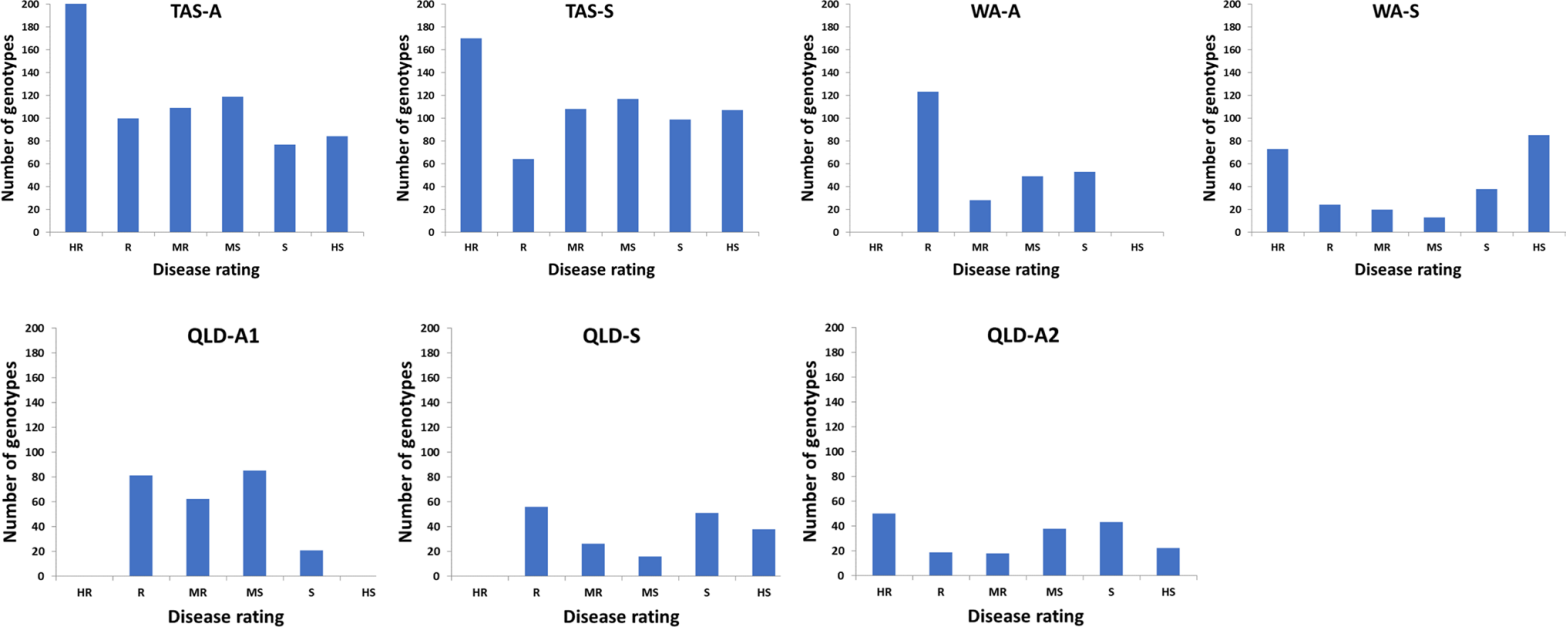
Distribution of powdery mildew resistance in a collection of 696 barley accession lines assessed in Tasmania (TAS), 254 barley accession lines assessed in Western Australia (WA) and Queensland (QLD). Two barley growth stages were assessed for powdery mildew resistance. A: barley adult stage; S: barley seedling stage. Six disease ratings were presented as HR: highly resistant; R: resistant; MR: moderately resistant; MS: moderately susceptible; S: susceptible; HS: highly susceptible. In QLD, adult stage assessments were conducted twice (A1 and A2)

Accessions selected for this experiment showed a wide variation in PM resistance (Fig. 1, Table S1). In Tasmanian seedling test trials, 201 genotypes showed complete resistance and 161 were highly susceptible to PM. For adult-plant resistance, 170 showed complete resistance and 107 were highly susceptible to PM. A total of 118 genotypes showed complete resistance in both seedling and adult-plant stages. Twenty-six genotypes showed intermediate levels of resistance in the adult-plant stage but were susceptible in the seedling stage, while 62 genotypes showed resistance in the seedling stage but were susceptible in the adult-plant stage.

In the WA trials, the resistance at the seedling and adult-plant stages was relatively consistent. All 73 of 253 genotypes showing complete seedling resistance were also resistant at the adult-plant stage. Similarly in the QLD trials, most genotypes that were resistant at the seedling stage showed good resistance at the adult-plant stage. Only a small number of genotypes showed resistance at the seedling stage yet were susceptible at the adult-plant stage.

**Fig. 2:**
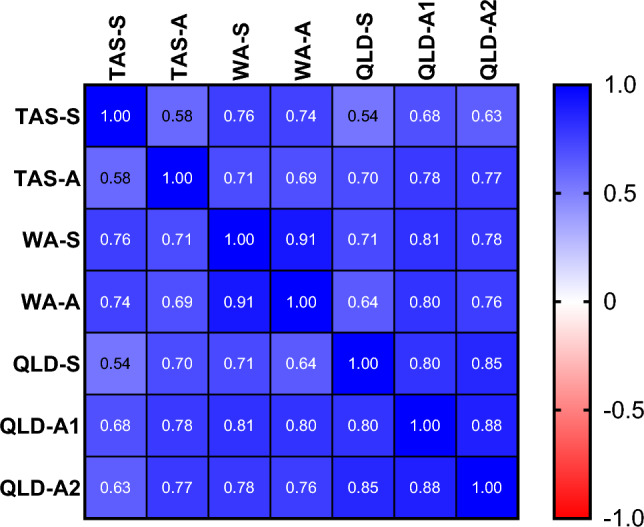
Correlation coefficients of powdery mildew resistance scores between different locations/stages

The correlation between seedling resistance and adult-plant resistance from the TAS trials was significant but the correlation coefficient (*r* = 0.58) was lower than those from the WA trials (*r* = 0.91) and the QLD trials (*r* = 0.85–0.88). Correlations between different sites for the seedling resistance were relatively moderate with *r*-values ranging from 0.5 to 0.7 (Fig. 2). The ANOVA showed significant differences among genotypes (Table S2–S4) with high heritability (*h*^2^ = 0.85 based on the TAS adult stage resistance which had the most numbers of genotypes). Significant interaction for genotypes and testing sites (Table S4) was also identified, which is reflected by the fact that some genotypes were highly resistant in one site but susceptible or highly susceptible in other sites.

### QTL identified from the genome-wide association study

The structure analysis on the GWAS population showed that there was no significant linkage among varieties (weak population structure), confirming the rationality of using this population. The FarmCPU model has a higher statistical power compared to both GLM and MLM models when evaluating populations with either weak or strong population structures (Yin et al. 2021). Quantile–quantile (Q-Q) plots (Fig. S1) showed that the FarmCPU model was better than the general linear model (GLM) and mixed linear model (MLM). Thus, the FarmCPU model was used for identifying marker-trait associations (MTAs), which identified 4–7 MTAs from different trials in this GWAS. Table 1 shows that seven significant MTAs were identified for the TAS seedling resistance with the major one on chromosome 1H (*P* = 6.8E−11); two on 2H (more likely a single QTL), one on 3H; two on 5H (more likely a single QTL) and one on 3H. For the TAS adult resistance, two major MTAs were found on 7H. Other relatively weaker MTAs were located on chromosomes 1H, 3H (2 MTAs), and 5H. For the WA seedling resistance, one major MTA was located on 1H and the other major one on 5H. Another three MTAs were located on chromosomes 2H, 5H, and 7H, respectively. Seven significant MTAs were detected for the WA adult-plant resistance. Two major ones were observed on 3H and 7H, and other MTAs were identified on 1H, 2H (2 MTAs), 5H, and 6H. The MTAs identified from the QLD trials were significant but with lower LOD values. Six significant MTAs for the QLD seedling resistance were located on 1H (2 MTAs), 3H, 4H, and 5H (2 MTAs). The six significant MTAs for the QLD adult-plant resistance were located on 1H (2 MTAs at similar positions), 2H, 3H, 6H, and 7H (Table 1).

**Table 1:** Chromosome positions of the markers showing significant associations with powdery mildew disease severity at different locations/sowing times.

Locations/ traits	Associated marker	Chr	Chromosome position (bp)	*P* value	Effect
TAS-A	3915937	1H	5,667,060	2.50E−06	− 0.19
	4196935	3H	21,372,691	2.67E−07	− 0.09
	7749489	3H	588,111,913	1.90E−06	− 0.16
	3263293	5H	597,382,003	2.22E−08	− 0.01
	36097751	7H	4,785,890	2.41E−10	0.26
	3930965	7H	10,868,513	1.01E−12	− 0.51
TAS-S	5257779	1H	3,910,732	6.83E−11	− 0.14
	3664807	2H	519,074,198	1.04E−07	0.08
	3274336	2H	653,225,321	3.79E−07	0.07
	3267803	3H	19,757,122	2.57E−06	− 0.10
	4184933	5H	513,428,293	1.58E−06	0.17
	3263522	5H	548,140,044	2.04E−06	0.18
	3265616	7H	10,055,290	1.41E−06	− 0.57
QLD-A1	5257779	1H	3,910,732	6.67E−07	− 0.15
	3262333	2H	565,112,217	1.25E−06	0.03
	5257372	3H	14,925,627	7.26E−07	− 0.06
	3274467	3H	518,111,158	2.28E−07	0.09
QLD-A2	3272378	1H	5,657,992	1.84E−06	− 0.09
	3930557	1H	10,804,735	8.74E−09	− 0.14
	3987258	2H	91,549,842	1.09E−09	0.09
	3257121	3H	627,523,088	1.08E−06	− 0.07
	5255918	6H	6,923,802	1.25E−07	− 0.02
	4006733	7H	12,249,254	2.58E−09	− 0.28
QLD-S	4789881	1H	7,434,321	1.21E−07	− 0.14
	3916677	1H	482,474,031	1.19E−07	− 0.20
	3663237	3H	577,221,407	1.15E−07	− 0.15
	5240930	4H	549,314,153	3.09E−09	0.15
	3811072	5H	6,573,035	9.02E−07	0.56
	3271753	5H	531,514,153	3.06E−07	0.07
WA-A	3260133	1H	8,027,708	2.26E−08	− 0.07
	3266784	2H	78,752,216	2.98E−07	− 0.16
	3258706	2H	617,065,134	8.70E−07	0.12
	3396532	3H	103,888,735	1.03E−11	0.16
	3663215	5H	111,483,505	1.97E−07	− 0.06
	5255918	6H	6,923,802	1.85E−08	0.09
	3432242	7H	17,049,713	9.81E−12	0.30
WA-S	3260133	1H	8,027,708	8.06E−20	− 0.12
	7243100	2H	38,122,301	2.19E−06	0.37
	3811072	5H	6,573,035	1.76E−06	0.25
	4017222	5H	570,723,506	5.07E−11	− 0.03
	3255623	7H	11,351,557	1.92E−08	− 0.10

*TAS* Tasmania, *QLD* Queensland, *WA* Western Australia, *A* adult stage resistance, *S* seedling stage resistance

**Fig. 3:**
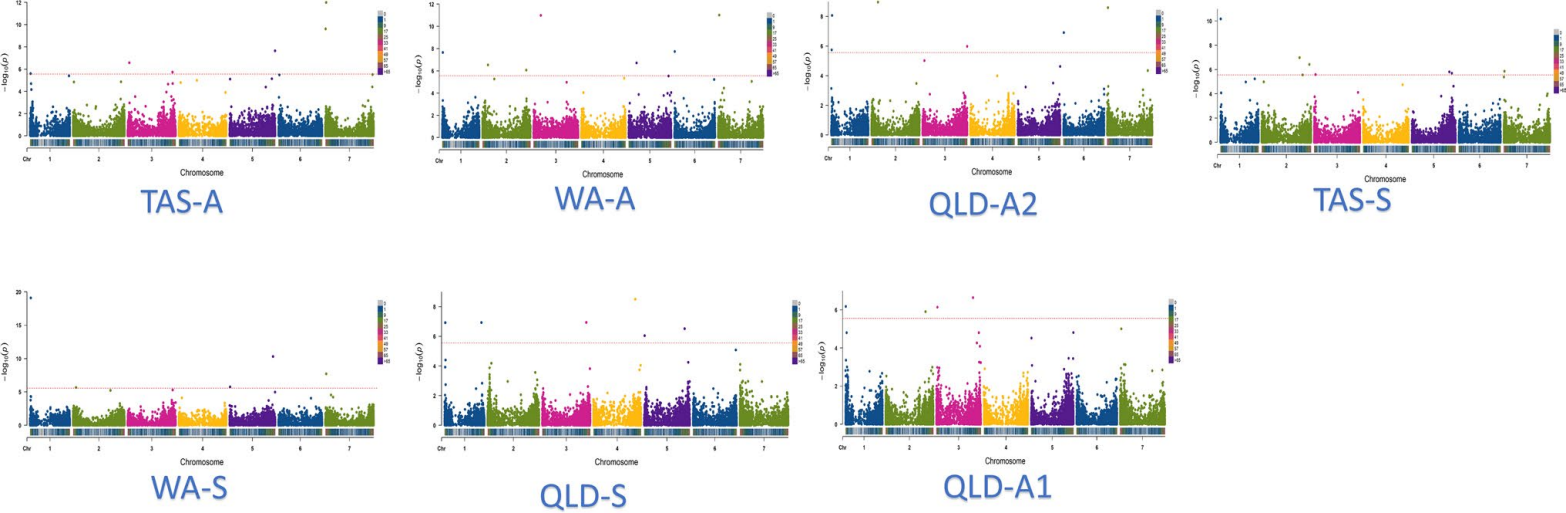
Manhattan plots using FarmCPU method for genome wide association study (GWAS) of powdery mildew resistance in barley at different locations and growth stages. Two barley growth stages were assessed for powdery mildew resistance. A: barley adult stage; S: barley seedling stage; TAS: Tasmania; QLD: Queensland; WA: Western Australia. In QLD, adult stage assessments were conducted twice (A1 and A2). A threshold of − log10(*P*) = 5.5 was applied

**Fig. 4:**
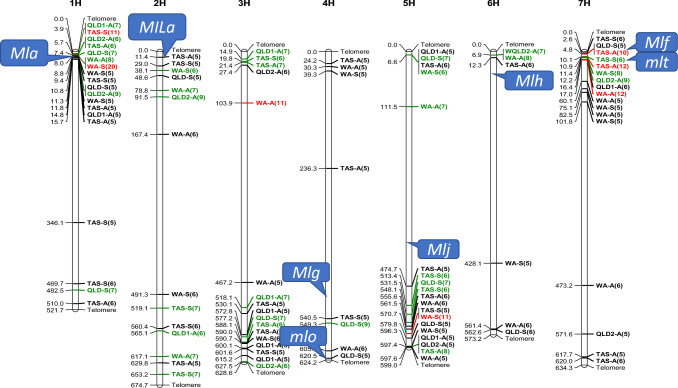
Genomic distribution of MTAs on barley chromosomes for powdery mildew resistance: strong MTAs are in green (*p* values between 10^–6^ and 10^–10^) and red font (*p* values less than 10^–10^); relatively weaker MTAs with *p* values between 10^–4^ and 10^–5^ are in black font. Based on vast literature studies, previously reported cloned genes were also highlighted and added to the corresponding chromosome positions

The distribution of all significant MTAs is presented in Figs. 3 and 4. Significant MTAs were found on all chromosomes with most of them being distributed on 1HS, 5HL, and 7HS. In Fig. 3, some significant MTAs with lower LOD values were also detected, most of these occurred in regions where the significant MTAs were located.

High protein sequence similarity typically corresponds to a comparable protein function. Following this strategy, we meticulously selected nine genes that have been cloned and functionally characterized for their involvement in imparting resistance to PM (Fig. S2). These genes either encode protein containing DNA/RNA binding domains (Zhou et al. 2001), involve RNA degradation for programmed cell death (Xi et al. 2009) or encode protein kinases and transcription factors at a signaling transduction scope for PM resistance (Rayapuram et al. 2012; Han et al. 2020). Through protein sequence blast against the whole genome, the genes falling within the identified QTL regions were specifically identified as candidate genes. In accordance with this strategy, five highly possible candidate genes (HORVU1Hr1G085870, HORVU1Hr1G051260, HORVU3Hr1G109350, HORVU3Hr1G073960, HORVU4Hr1G031590) distributed on 1HL, 3HL and 4H situated within the QTL region were proposed. Additional elaboration on these findings has been included in the discussion section.

### Cumulative effects of alleles on disease response

**Fig. 5:**
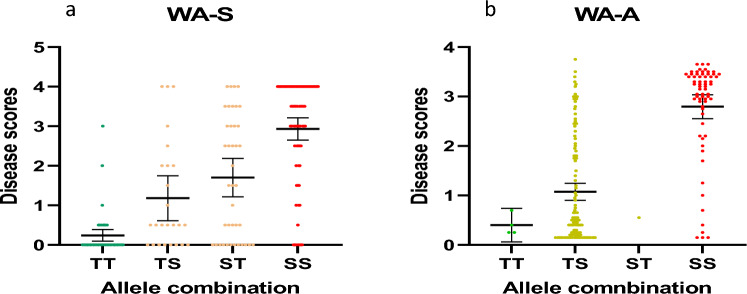
Distribution of disease scores of genotypes with different combinations of resistant alleles of two most significant MTAs. SS = sensitive alleles from both MTAs, TS/ST = resistant allele from one MTA and sensitive allele from the other MTA, TT = resistant alleles from both MTAs. **a**: seedling stage resistance from WA trial predicted from 3260133 on 1H and 4017222 on 5H; **b**: adult-plant stage resistance from WA trial predicted from 3432242 on 7H and 3663215 on 5H. The bars in the figure indicate the average values and 95% confidence intervals performed using GraphPad Prism 10

Most of the MTAs showed additive effects on the resistance. Figure 5a shows an example of seedling resistance from the WA trial. The two most significant MTAs (3260133 on 1H and 4017222 on 5H) were highly effective on the seedling resistance. Where genotypes carried both resistance alleles (TT), the average seedling disease score was 0.24 ± 0.07. This contrasted with genotypes that carried only one resistance allele (TS or ST), where the average seedling disease scores were 1.18 ± 0.27 and 1.70 ± 0.24, respectively. The average seedling disease score of genotypes carrying both susceptibility alleles (SS) was 2.93 ± 0.14. Most importantly, of all the genotypes in the TT group, only one showed susceptibility, indicating the efficiency of using these two markers to select resistant lines. In the SS group, there were still some lines showing complete resistance, suggesting the existence of other resistance QTL/genes. Similarly for the adult-plant resistance in the WA trial, the two most significant MTAs (3432242 on 7H and 3663215 on 5H) also showed their effectiveness on the adult-plant resistance. The average scores for genotypes TT, TS, ST and SS were 0.40 ± 0.11, 1.07 ± 0.09, 0.55 (only one genotype) and 2.79 ± 0.12, respectively (Fig. 5b).

## Discussion

Many barley varieties grown in Australia remain susceptible to powdery mildew at regional and national levels. It is a devastating disease that affects whole crop growth from seedling to adult-plant stages. At the adult-plant stage, even under a low level of infection (4% leaf area diseased), both assimilation and transpiration rates are considerably reduced (Rabbinge et al. 1985). Furthermore, the severity of mildew infection is highly linked with host water and nitrogen contents, suggesting higher levels of mildew infection in well-irrigated and fertilized fields, increasing the need for crop management at adult-plant stages (Thompson et al. 1993). While damage from PM at seedling stages cannot be overlooked, damage at adult-plant stages should be paid even greater attention since plant health at the adult-plant stages fundamentally decides crop yield and quality (Ge et al. 2021).

Most of the current research to detect novel sources of disease resistance using GWAS is based on disease screening from a limited number of environments and consistent performance of resistance genes/loci/QTL under different geographic climates is mostly overlooked (Ames et al. 2015). In this study, candidate germplasm was screened in three states of Australia (Tasmania, Western Australia, and Queensland) each with very different climates and potentially different pathotypes of the pathogen (Dreisetl et. al. 2013). Significant variation in PM resistance was found not only between seedling and adult-plant stages but also across different states. However, similar QTL regions were identified from the screening data from each state and at different growth stages with some variation in the contribution of different major QTL and the detection of minor QTL (Fig. 3). For example, significant MTAs for the adult-plant PM resistance were identified on chromosomes 1H, 3H, 5H, and 7H and significant MTAs for the seedling resistance were identified on 5H and 7H from all three states (Fig. 3). Some relatively weaker MTAs were identified in only one state or at one specific growth stage (Table 1) with a major QTL region for the adult-plant resistance on 3H identified in WA only. These findings confirmed the presence of previously reported QTL/genes that were distributed across all barley chromosomes.

Accumulative research has reported QTL/genes responsible for PM resistance by default at the seedling stage. Among them, *Mla12* is one of the major ones for PM resistance at the seedling stages (Freialdenhoven et al. 1994). In some cases, no crop growth stage is given suggesting that barley varieties resistant to PM at the seedling stage maintain similar resistance to PM at adult-plant stages (Ames et al. 2015; Büschges et al. 1997; Wei et al. 1999). However, our research clearly illustrated the difference in resistance to powdery mildew between barley seedling and adult-plant stages (Figs. 1 and 3) with many seedling-resistant genotypes showing susceptibility at adult-plant stages or vice versa.

Genes underlying barley powdery mildew resistance have been functionally studied and reported on all chromosomes except 3H (Hickey et al. 2012; Piechota et al. 2019; Silvar et al. 2010). Representative genes that have been cloned are summarized in Fig. 4. Most reported QTL/cloned genes overlapped with MTAs identified from our research (Fig. 4). *Mla* was once clustered within a 240-Kb DNA interval on chromosome 1H (Wei et al. 1999), with *Mla12* identified as the major candidate gene for PM resistance in this region (Shen et al. 2003). It was demonstrated that *Mla* resistance to PM infection triggered a rapid response of overexpression of protein *Mla12* (Shen et al. 2003). Our results re-highlighted the importance of *Mla* with a cluster of MTAs being identified in the *Mla* region. Chromosome 1HS also contains several resistance genes ordered from the centromere: *Mlnn*, *Mlk*, *Mla6*, and *Mlra* (https://wheat.pw.usda.gov/GG3/BarleyBinMaps) (Ge et al. 2021). Other major genes, *MlLa*, *mlo*, and *Mlf/mlt* on 2H, 4H, and 7H, respectively, were all identified in our research (Fig. 4).

Growth stage dependent resistance to barley powdery mildew has been considered as partial resistance or complete resistance at adult-plant stages (Hwang and Heitefuss 1982; Mastebroek and Balkema-Boomstra 1991). A form of basal resistance was claimed as non-race-specific and durable resistance by Aghnoum et al. (2010), while Collins et al. (2003) described *mlo* and non-host resistance. The results of this study indicate that the above resistance genes may be present in the candidate lines as some QTL identified are close to these loci reported to be associated with these resistances (Fig. 4). For the *Mla* region on 1H, 15 MTAs (across three stages responsible for both seedling stage and adult stage) were found in our research (Fig. 4). While most QTL regions identified in this study overlapped with previously reported loci, our research also detected MTAs in distinct chromosomal positions, indicating potentially new genes for barley powdery mildew resistance (Fig. 4). A major MTA for the adult-plant PM resistance on 3HS was detected from the WA trial. It is to notice that Novakazi et al. (2020) also detected QTL at a similar position but responsible for seedling stage resistance. Our result raised the novelty that this QTL may also contribute to adult stage PM resistance. MTAs were also detected on 3HL from the TAS and QLD trials while a significant QTL region on 5HL was identified from all the sites. The positions of these MTAs are some distance away from the reported *Mlj* locus. The 5HL QTL region was shown to be important to both seedling and adult-plant resistance selection (Fig. 5). These new QTL need to be validated through analysis of segregating populations.

These MTAs identified on 7HS overlapped with the qualitative resistance gene *Mlf*, consistent with previous research in QTL mapping where marker EBmac0755 was associated and located in a similar position (Silvar et al. 2010). *Mlf* has been described in the wild barley *H. spontaneum* for powdery mildew resistance. *Mlt* is another resistance gene identified as race specific. *Mlt* was also mapped to the short arm of chromosome 7H, associated with SSR marker *GBM1060*, derived from *H. spontaneum* (Silvar et al. 2010). However, whether *Mlf* and *Mlt* show partial or complete resistance to powdery mildew at the adult-plant stage is not clearly stated (Schönfeld et al. 1996). *Rbgq20* on 7HS at a similar position to *Mlt* was effective in seedling leaves only (Aghnoum et al. 2010). However, in another study following a pathotype screening, a major QTL on 7HS (named *Rbgh2*) played a significant role in controlling adult-plant resistance (Ge et al. 2021). A QTL on 7HS was identified based on seedling phenotyping in the Spanish landrace SBCC097, explaining up to 18.5% of the variance (Silvar et al. 2010). All these findings support the high density of MTAs identified at the top region of 7H from our research. Our research showed that the QTL region on 7HS contributed mainly to the adult-plant resistance while the QTL on 1HS made significant contributions to seedling resistance. As shown in Fig. 4, the combination of MTAs on 1HS and 5HL could not only deliver superior seedling resistance but also impact more on the adult-plant resistance.

Proteins that have similar sequences tend to present similar functions for PM resistance. A representative example is the identification of gene *Mla12* on barley chromosome 1H. *Mla12* encodes a protein that shares 89% and 92% identical amino acids with the known proteins MLA1 and MLA6, which are identified as genes specifically for barley PM resistance (Shen et al. 2003). Based on this strategy, for accurately identifying putative novel genes underpinning barley PM resistance, we searched genes within the identified QTL regions that share high similarity in sequence with representative genes reported for powdery mildew resistance. We selected nine representative genes that have been cloned and their functions in regulating barley powdery mildew have been studied. For example, barley *Mla* locus confers multiple resistance specificities to powdery mildew and was mapped on 1HS, encoding a 108-Kd protein containing an N-terminal coiled-coil structure, a central nucleotide binding domain, and a C-terminal leucine-rich repeat region (Zhou et al. 2001). Overexpression of barley *mla1* in wheat also induced resistance to wheat powdery mildew, indicating evolutionary conservation of *mla1* in two closely linked homeoloci (Zhou et al. 2001). Having a high similarity in protein sequences with *mla1*, *mla6* is another cloned gene for powdery mildew resistance (Halterman et al. 2001). Due to the high similarity in protein sequence of Mla1 and Mla6, one region on 2HS and the other one on 5HL were found (Fig. S2). These two regions were co-located with positions of genes *MILa* on 2H and *Mlj* on 5H (Fig. 4). RRP46 encodes a critical component mediating RNA processing and degradation involved in programmed cell death initiation as a result of resistance to barley powdery mildew resistance (Xi et al. 2009). Blastp (protein sequence blast) results of RRP46 indicated a candidate gene (*HORVU1Hr1G085870*) located on 1HL, overlapping identified MTA [TAS-A(6)] in our study (Fig. 4). SnRK1 (sucrose non-fermenting-related kinase1) and WRKY3 (transcription factor) play roles in barley powdery mildew resistance (Han et al. 2020). Overexpressing WRKY3 enhances fungal microcolony formation and sporulation, thus decreasing resistance to powdery mildew. The overexpression of SnRK1 physically interacts with WRK3 through phosphorylation and destabilization, reducing fungal haustorium formation in barley cells (Han et al. 2020). Protein sequence blast from SnRK1 and WRKY3 both presented putative candidate genes on 1H but at different genomic regions (Fig. S2). Specifically, the WRK3 blast result indicated a novel candidate gene (*HORVU1Hr1G051260*) within 1H QTL [TAS-S(5)] (Figs. S2 and 4). Barley receptor-like protein kinases (RLKs) control numerous plant physiological processes, including development, hormone perception, and stress responses. The cysteine-rich RLKs (CRKs) represent a prominent subfamily of transmembrane-anchored RLKs. *HvCRK1* belongs to this gene family, localizes to the endoplasmic reticulum, and plays significant roles in regulating barley powdery mildew resistance (Rayapuram et al. 2012). HvCRK1 blast result suggested a novel candidate gene *HORVU3Hr1G109350* on 3HL, coincident with identified QTL on this chromosome (Figs. 4 and S2). Blast results from SnRK1 (Han et al. 2020), *HvSERK2* (Somatic embryogenesis receptor-like kinase) (Li et al. 2018), and HvGsl6 (glucan synthase-like 6) (Chowdhury et al. 2016) all indicated another candidate gene, *HORVU3Hr1G073960*, overlapping the MTA (WA-A), on 3H (Fig. 4). HvRBK1 (Binding protein kinase that interacts with receptor-like cytoplasmic kinase) (Huesmann et al. 2012) is another cloned gene for barley powdery mildew resistance. The protein blast result from HvRBK1 indicated a novel candidate gene, *HORVU4Hr1G031590*, located on 4H, coincident with an MTA of TAS-A(5) on the same chromosome.

In conclusion, the barley lines containing effective MTAs are a useful resource for any breeding program. Multiple MTAs for seedling and adult-plant PM resistance were identified. The role of the 3HS QTL region on adult plant resistance was not previously reported and therefore needs to be studied further to assess its potential for use in breeding programs. This study also demonstrated that the QTL region on 7HS was responsible for adult-plant resistance while the one on 1HS was mainly responsible for the seedling resistance. As resistance to powdery mildew is required at all stages of crop development, pyramiding favorable alleles of different QTL that function at both seedling and\or adult-plant growth stages is required to produce high levels of durable resistance.

### Future perspective

In response to the pathogen's ability to adapt to the environment and overcome gene-to-gene resistance, the continuous exploration of new genes for PM resistance is imperative. Furthermore, considering the pathogen's localized nature, the identification of location-specific genes/QTL conferring resistance to PM in specific locations/zones is particularly relevant for agricultural practices within the scope of our research findings. Building upon the proposed candidate genes, the development of specific gene markers holds potential for: (1) assessing the contribution of these genes to PM resistance, and (2) expediting the crop breeding process by facilitating the breeding of PM-resistant crops. Future investigations should also prioritize gene mapping and the pyramiding of QTL regions associated with robust seedling and adult stage resistance.

### Supplementary Information

Below is the link to the electronic supplementary material.

Supplementary file1 (PPTX 572 KB)

Supplementary file2 (XLSX 62 KB)

Supplementary file3 (DOCX 17 KB)

## Data Availability

The datasets supporting the conclusions of this article and specific germplasm can be obtained from the corresponding author, Prof Meixue Zhou, TIA, University of Tasmania, under Material Transfer Agreement.

## References

[CR1] Aghnoum R, Marcel TC, Johrde A, Pecchioni N, Schweizer P, Niks RE (2010). Basal host resistance of barley to powdery mildew: connecting quantitative trait loci and candidate genes. Mol Plant Microbe Interact.

[CR2] Alqudah AM, Sallam A, Baenziger PS, Börner A (2020). GWAS: fast-forwarding gene identification and characterization in temperate cereals: lessons from barley—a review. J Adv Res.

[CR3] Ames N, Dreiseitl A, Steffenson BJ, Muehlbauer GJ (2015). Mining wild barley for powdery mildew resistance. Plant Pathol.

[CR4] Brent K, Carter G, Hollomon D, Hunter T, Locke T, Proven M (1989). Factors affecting build-up of fungicide resistance in powdery mildew in spring barley. Neth J Plant Pathol.

[CR5] Büschges R, Hollricher K, Panstruga R, Simons G, Wolter M, Frijters A, van Daelen R, van der Lee T, Diergaarde P, Groenendijk J (1997). The barley *Mlo* gene: a novel control element of plant pathogen resistance. Cell.

[CR6] Chowdhury J, Schober MS, Shirley NJ, Singh RR, Jacobs AK, Douchkov D, Schweizer P, Fincher GB, Burton RA, Little A (2016). Down-regulation of the glucan synthase-like 6 gene (*HvGsl6*) in barley leads to decreased callose accumulation and increased cell wall penetration by *Blumeria graminis* f. sp. *hordei*. New Phytol.

[CR7] Chu W, Li R, Liu J, Reimherr M (2020). Feature selection for generalized varying coefficient mixed-effect models with application to obesity GWAS. Ann Appl Stat.

[CR8] Collins NC, Thordal-Christensen H, Lipka V, Bau S, Kombrink E, Qiu J-L, Hückelhoven R, Stein M, Freialdenhoven A, Somerville SC (2003). SNARE-protein-mediated disease resistance at the plant cell wall. Nature.

[CR9] Cowger C, Mehra L, Arellano C, Meyers E, Murphy JP (2018). Virulence differences in *Blumeria graminis* f. sp*. tritici* from the central and eastern United States. Phytopathology.

[CR10] Dreiseitl A (2017). Genes for resistance to powdery mildew in European barley cultivars registered in the Czech Republic from 2011 to 2015. Plant Breed.

[CR11] Dreiseitl A (2020). Specific resistance of barley to powdery mildew, its use and beyond a concise critical review. Genes (basel).

[CR12] Dreiseitl A (2022). Postulation of specific disease resistance genes in cereals: a widely used method and its detailed description. Pathogens.

[CR13] Dreiseitl A, Fowler RA, Platz GJ (2013). Pathogenicity of *Blumeria graminis* f. sp. *hordei* in Australia in 2010 and 2011. Australas Plant Pathol.

[CR14] Flor HH (1971). Current status of the gene-for-gene concept. Annu Rev Phytopathol.

[CR15] Freialdenhoven A, Scherag B, Hollricher K, Collinge DB, Thordal-Christensen H, Schulze-Lefert P (1994). *Nar-1* and *Nar-2*, two loci required for *Mla12*-specified race-specific resistance to powdery mildew in barley. Plant Cell.

[CR16] Ge C, Wentzel E, D'Souza N, Chen K, Oliver RP, Ellwood SR (2021). Adult resistance genes to barley powdery mildew confer basal penetration resistance associated with broad-spectrum resistance. Plant Genome.

[CR17] Golzar H, Shankar M, D'Antuono M (2016). Responses of commercial wheat varieties and differential lines to western Australian powdery mildew (*Blumeria graminis* f. sp *tritici*) populations. Australas Plant Pathol.

[CR18] Gupta S, Vassos E, Sznajder B, Fox R, Khoo KH, Loughman R, Chalmers KJ, Mather DE (2018). A locus on barley chromosome 5H affects adult plant resistance to powdery mildew. Mol Breed.

[CR19] Halterman D, Zhou F, Wei F, Wise RP, Schulze-Lefert P (2001). The MLA6 coiled-coil, NBS-LRR protein confers AvrMla6-dependent resistance specificity to *Blumeria graminis* f. sp. *hordei* in barley and wheat. Plant J.

[CR20] Han X, Zhang L, Zhao L, Xue P, Qi T, Zhang C, Yuan H, Zhou L, Wang D, Qiu J (2020). SnRK1 phosphorylates and destabilizes WRKY3 to enhance barley immunity to powdery mildew. Plant Comm.

[CR21] Hickey LT, Lawson W, Platz GJ, Fowler RA, Arief V, Dieters M, Germán S, Fletcher S, Park RF, Singh D (2012). Mapping quantitative trait loci for partial resistance to powdery mildew in an Australian barley population. Crop Sci.

[CR22] Huesmann C, Reiner T, Hoefle C, Preuss J, Jurca ME, Domoki M, Fehér A, Hückelhoven R (2012). Barley ROP binding kinase1 is involved in microtubule organization and in basal penetration resistance to the barley powdery mildew fungus. Plant Physiol.

[CR23] Hwang B, Heitefuss R (1982). Characterization of adult plant resistance of spring barley to powdery mildew (*Erysiphe graminis* f. sp. *hordei*). II. Infection process at different leaf stages. J Phytopathol.

[CR24] Jørgensen IH (1992). Discovery, characterization and exploitation of *Mlo* powdery mildew resistance in barley. Euphytica.

[CR25] Jørgensen JH (1994). Genetics of powdery mildew resistance in barley. Crit Rev Plant Sci.

[CR26] Kaler AS, Gillman JD, Beissinger T, Purcell LC (2020). Comparing different statistical models and multiple testing corrections for association mapping in soybean and maize. Front Plant Sci.

[CR27] Kusmec A, Schnable PS (2018). FarmCPUpp: efficient large-scale genomewide association studies. Plant Direct.

[CR28] Li H, Zhou M (2011). Quantitative trait loci controlling barley powdery mildew and scald resistances in two different barley doubled haploid populations. Mol Breed.

[CR29] Li Y, Li Q, Guo G, He T, Gao R, Faheem M, Huang J, Lu R, Liu C (2018). Transient overexpression of *HvSERK2* improves barley resistance to powdery mildew. Int J Mol Sci.

[CR30] Mastebroek H, Balkema-Boomstra A (1991). Identification of growth stage dependent expression of partial resistance of barley to powdery mildew. Euphytica.

[CR31] Monat C, Padmarasu S, Lux T, Wicker T, Gundlach H, Himmelbach A, Ens J, Li C, Muehlbauer GJ, Schulman AH, Waugh R, Braumann I, Pozniak C, Scholz U, Mayer KFX, Spannagl M, Stein N, Mascher M (2019). TRITEX: chromosome-scale sequence assembly of Triticeae genomes with open-source tools. Genome Biol.

[CR32] Novakazi F, Krusell L, Jensen JD, Orabi J, Jahoor A, Bengtsson T (2020). PPP Barley Consortium (2020) You had me at “MAGIC”!: four barley MAGIC populations reveal novel resistance QTL for powdery mildew. Genes.

[CR33] Oerke E-C, Dehne H-W, Schönbeck F, Weber A (1999). Crop production and crop protection: estimated losses in major food and cash crops.

[CR34] Piechota U, Czembor PC, Słowacki P, Czembor JH (2019). Identifying a novel powdery mildew resistance gene in a barley landrace from Morocco. J Appl Genet.

[CR35] Piechota U, Słowacki P, Czembor PC (2020). Identification of a novel recessive gene for resistance to powdery mildew (*Blumeria graminis* f. sp. *hordei*) in barley (*Hordeum vulgare*). Plant Breed.

[CR36] Rabbinge R, Jorritsma I, Schans J (1985). Damage components of powdery mildew in winter wheat. Neth J Plant Pathol.

[CR37] Rayapuram C, Jensen MK, Maiser F, Shanir JV, Hornshøj H, Rung JH, Gregersen PL, Schweizer P, Collinge DB, Lyngkjaer MF (2012). Regulation of basal resistance by a powdery mildew-induced cysteine-rich receptor-like protein kinase in barley. Mol Plant Pathol.

[CR38] Razzaq A, Wani SH, Saleem F, Yu M, Zhou M, Shabala S (2021). Rewilding crops for climate resilience: economic analysis and *de novo* domestication strategies. J Exp Bot.

[CR39] Schönfeld M, Ragni A, Fischbeck G, Jahoor A (1996). RFLP mapping of three new loci for resistance genes to powdery mildew (*Erysiphe graminis* f. sp. *hordei*) in barley. Theor Appl Genet.

[CR40] Shen Q-H, Zhou F, Bieri S, Haizel T, Shirasu K, Schulze-Lefert P (2003). Recognition specificity and RAR1/SGT1 dependence in barley *Mla* disease resistance genes to the powdery mildew fungus. Plant Cell.

[CR41] Shtaya M, Marcel T, Sillero JC, Niks RE, Rubiales D (2006). Identification of QTLs for powdery mildew and scald resistance in barley. Euphytica.

[CR42] Silvar C, Dhif H, Igartua E, Kopahnke D, Gracia MP, Lasa JM, Ordon F, Casas AM (2010). Identification of quantitative trait loci for resistance to powdery mildew in a Spanish barley landrace. Mol Breed.

[CR43] Thompson G, Brown J, Woodward F (1993). The effects of host carbon dioxide, nitrogen and water supply on the infection of wheat by powdery mildew and aphids. Plant Cell Environ.

[CR01] Torp J, Jensen HP, Jørgensen JH (1978) Powdery mildew resistance genes in 106 Northwest European spring barley varieties. Kongelige Veterinaer- og Landbohoejskole. Aarskrift, 75–102

[CR44] Van Ooijen JW (2009). MapQTL 6.0, software for the mapping of quantitative trait loci in experimental populations of dihaploid species.

[CR45] Wei F, Gobelman-Werner K, Morroll SM, Kurth J, Mao L, Wing R, Leister D, Schulze-Lefert P, Wise RP (1999). The *Mla* (powdery mildew) resistance cluster is associated with three NBS-LRR gene families and suppressed recombination within a 240-kb DNA interval on chromosome 5S (1HS) of barley. Genetics.

[CR46] Xi L, Moscou MJ, Meng Y, Xu W, Caldo RA, Shaver M, Nettleton D, Wise RP (2009). Transcript-based cloning of RRP46, a regulator of rRNA processing and R gene–independent cell death in barley-powdery mildew interactions. Plant Cell.

[CR47] Yin L, Zhang H, Tang Z, Xu J, Yin D, Zhang Z, Yuan X, Zhu M, Zhao S, Li X (2021). rmvp: A memory-efficient, visualization-enhanced, and parallel-accelerated tool for genome-wide association study. Genom Proteom Bioinform.

[CR02] Zadoks JC, Chang TT, Konzak CF (1974). A decimal code for the growth stages of cereals. Weed Res.

[CR48] Zhou F, Kurth J, Wei F, Elliott C, Valè G, Yahiaoui N, Keller B, Somerville S, Wise R, Schulze-Lefert P (2001). Cell-autonomous expression of barley *Mla1* confers race-specific resistance to the powdery mildew fungus via a Rar1-independent signaling pathway. Plant Cell.

