# Supplementary material for: Genome-wide association mapping for seedling and adult resistance to powdery mildew in barley
Source: Theor Appl Genet. 2024 Feb 16;137(3):50. doi: 10.1007/s00122-024-04550-y (PMC10873221; doi:10.1007/s00122-024-04550-y)
Supplement: Supplementary file 1 — Supplementary file1 (PPTX 572 KB) [file 122_2024_4550_MOESM1_ESM.pptx]

## Slide 1
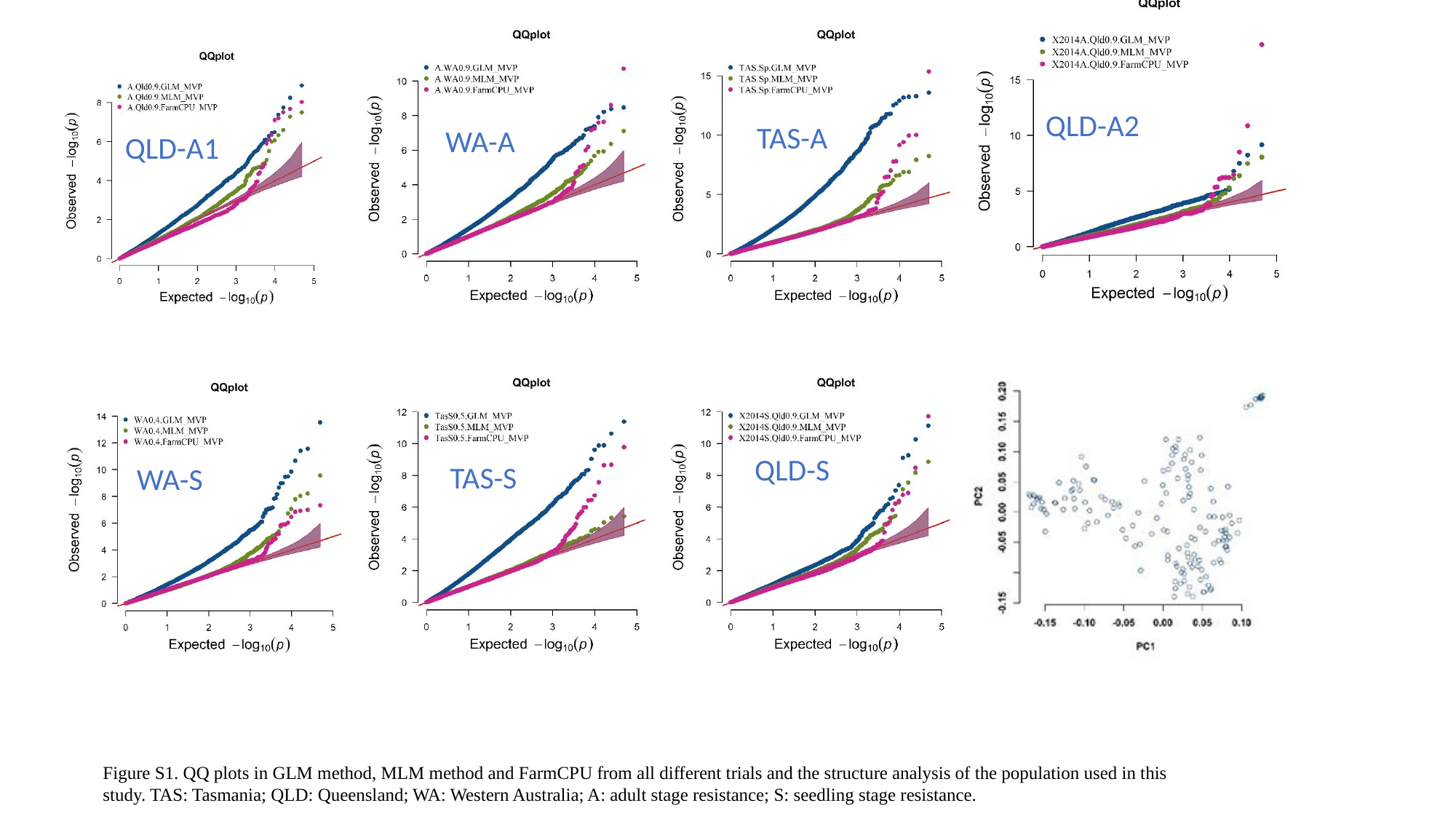

QLD-A2
TAS-A
WA-A
QLD-A1
QLD-S
TAS-S
WA-S
Figure S1. QQ plots in GLM method, MLM method and FarmCPU from all different trials and the structure analysis of the population used in this study. TAS: Tasmania; QLD: Queensland; WA: Western Australia; A: adult stage resistance; S: seedling stage resistance.

## Slide 2
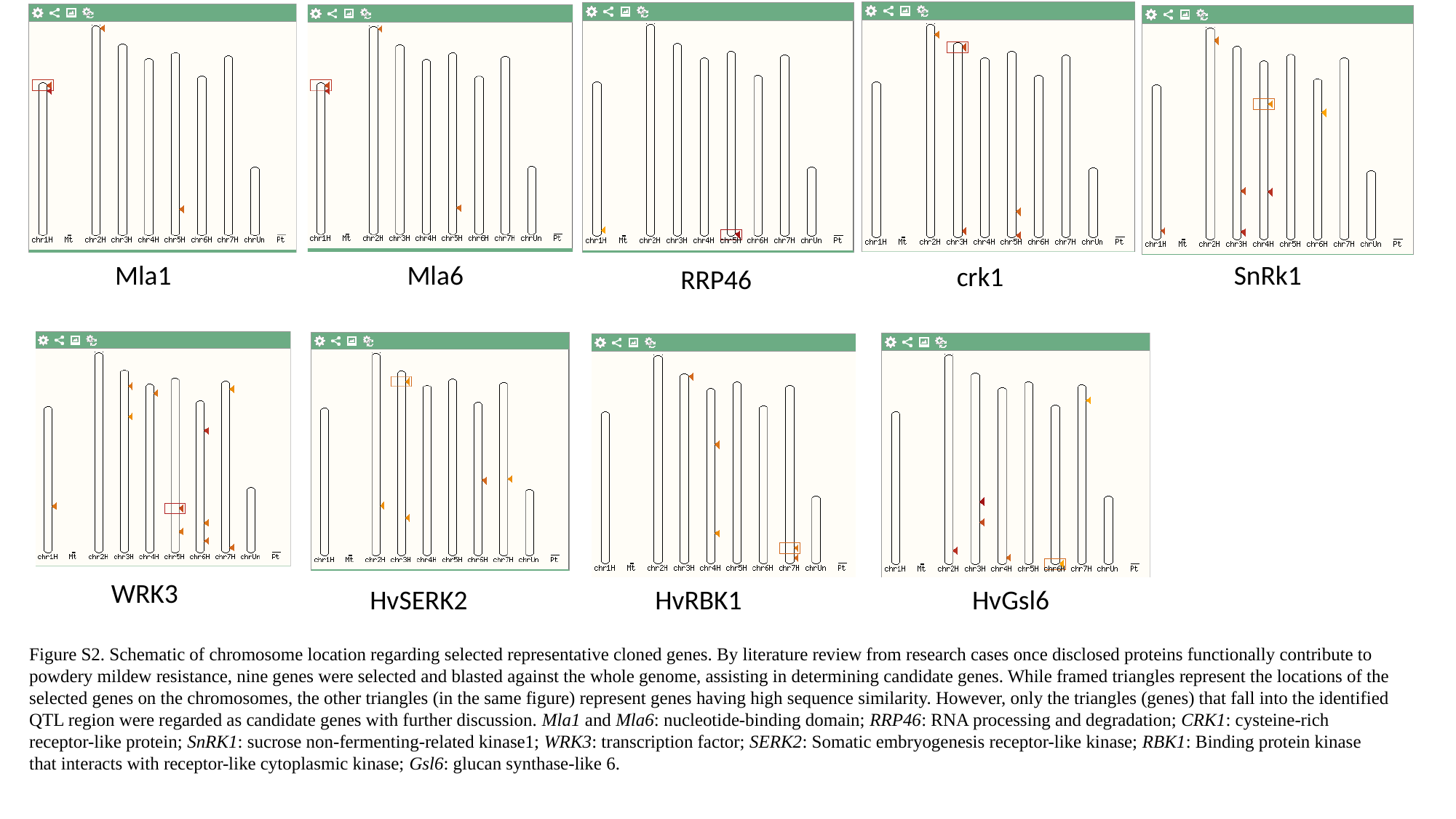

Mla1
Mla6
SnRk1
crk1
RRP46
WRK3
HvSERK2
HvRBK1
HvGsl6
Figure S2. Schematic of chromosome location regarding selected representative cloned genes. By literature review from research cases once disclosed proteins functionally contribute to powdery mildew resistance, nine genes were selected and blasted against the whole genome, assisting in determining candidate genes. While framed triangles represent the locations of the selected genes on the chromosomes, the other triangles (in the same figure) represent genes having high sequence similarity. However, only the triangles (genes) that fall into the identified QTL region were regarded as candidate genes with further discussion. Mla1 and Mla6: nucleotide-binding domain; RRP46: RNA processing and degradation; CRK1: cysteine-rich receptor-like protein; SnRK1: sucrose non-fermenting-related kinase1; WRK3: transcription factor; SERK2: Somatic embryogenesis receptor-like kinase; RBK1: Binding protein kinase that interacts with receptor-like cytoplasmic kinase; Gsl6: glucan synthase-like 6.
