# Supplementary material for: Genome-wide association mapping for seedling and adult resistance to powdery mildew in barley
Source: Theor Appl Genet. 2024 Feb 16;137(3):50. doi: 10.1007/s00122-024-04550-y (PMC10873221; doi:10.1007/s00122-024-04550-y)
Supplement: Supplementary file 3 — Supplementary file3 (DOCX 17 KB) [file 122_2024_4550_MOESM3_ESM.docx]

Table S2. ANOVA of seedling powdery mildew resistance from TAS

| Source of variation | Sum of Squares | DF | Mean Squares | F Value | P value |
| --- | --- | --- | --- | --- | --- |
| Total | 2458 | 882 |  |  |  |
| Replication | 5.46 | 1 | 5.46 | 6.10 | 0.014 |
| Genotype | 2257 | 662 | 3.41 | 3.81 | <0.001 |
| Error | 196 | 219 | 0.895 |  |  |

Table S3. ANOVA of adult powdery mildew resistance from TAS

| Source of variation | Sum of Squares | DF | Mean Squares | F Value | P value |
| --- | --- | --- | --- | --- | --- |
| Total | 4412 | 1355 |  |  |  |
| Replication | 1.52 | 1 | 1.52 | 3.06 |  |
| Genotype | 4110 | 688 | 5.97 | 12.06 | <0.001 |
| Error | 330 | 666 | 0.496 |  |  |

Table S4. ANOVA of powdery mildew resistance from three different sites

| Source of variation | Sum of Squares | DF | Mean Squares | F Value | P value |
| --- | --- | --- | --- | --- | --- |
| Total | 3249.3 | 1336 |  |  |  |
| Treatment | 2668.4 | 572 | 4.66 | 6.13 | <0.001 |
| Genotype | 2115.9 | 190 | 11.14 | 14.64 | <0.001 |
| Site | 187.0 | 2 | 93.49 | 122.95 | <0.001 |
| Genotype x Site | 365.5 | 380 | 0.96 | 1.26 | <0.05 |
| Error | 581.0 | 764 | 0.76 |  |  |
